# A Master Regulator of Bacteroides thetaiotaomicron Gut Colonization Controls Carbohydrate Utilization and an Alternative Protein Synthesis Factor

**DOI:** 10.1128/mBio.03221-19

**Published:** 2020-01-28

**Authors:** Guy E. Townsend, Weiwei Han, Nathan D. Schwalm, Xinyu Hong, Natasha A. Bencivenga-Barry, Andrew L. Goodman, Eduardo A. Groisman

**Affiliations:** a Department of Microbial Pathogenesis, Yale School of Medicine, New Haven, Connecticut, USA; b Yale Microbial Sciences Institute, West Haven, Connecticut, USA; c Department of Biochemistry and Molecular Biology, Penn State Health Milton S. Hershey Medical Center, Penn State Hershey College of Medicine, Hershey, Pennsylvania, USA; Brigham and Women’s Hospital/Harvard Medical School

**Keywords:** BT4338, EF-G2, *fusA2*, microbiota

## Abstract

Microbial colonization of the mammalian gut is largely ascribed to the ability to utilize nutrients available in that environment. To understand how beneficial microbes establish a relationship with their hosts, it is crucial to determine what other abilities promote gut colonization. We now report that colonization of the murine gut by the beneficial microbe Bacteroides thetaiotaomicron requires activation of a putative translation factor by the major transcriptional regulator of gut colonization and carbohydrate utilization. To ascertain how this regulator—called BT4338—promotes gut colonization, we identified BT4338-regulated genes and BT4338-bound DNA sequences. Unexpectedly, the gene whose expression was most reduced upon *BT4338* inactivation was *fusA2*, specifying a putative translation factor. We determined that *fusA2* activation by BT4338 is conserved in another *Bacteroides* species and essential for gut colonization in B. thetaiotaomicron because a mutant lacking the BT4338 binding site in the *fusA2* promoter exhibited a colonization defect similar to that of a mutant lacking the *fusA2* gene. Furthermore, we demonstrated that BT4338 promotes gut colonization independently of its role in carbohydrate utilization because the *fusA2* gene was dispensable for utilization of carbohydrates that depend on *BT4338*. Our findings suggest that microbial gut colonization requires the use of alternative protein synthesis factors.

## Abstract (precis)

The bacteria occupying the mammalian gut have evolved unique strategies to thrive in their environment. *Bacteroides* organisms, which often comprise 25 to 50% of the human gut microbiota, derive nutrients from structurally diverse complex polysaccharides, commonly called dietary fibers. This ability requires an expansive genetic repertoire that is coordinately regulated to achieve expression of those genes dedicated to utilizing only those dietary fibers present in the environment. Here we identify the global regulon of a transcriptional regulator necessary for dietary fiber utilization and gut colonization. We demonstrate that this transcription factor regulates hundreds of genes putatively involved in dietary fiber utilization as well as a putative translation factor dispensable for growth on such nutrients but necessary for survival in the gut. These findings suggest that gut bacteria coordinate cellular metabolism with protein synthesis via specialized translation factors to promote survival in the mammalian gut.

## INTRODUCTION

The gut microbiota is a key contributor to human health and development. Symbiotic microbes in the gut benefit their hosts by supplying essential nutrients, facilitating energy extraction from food, hindering pathogen invasion, and shaping host immunity (1–4). The host’s diet controls the composition of the gut microbiota by providing nutrients utilized only by those microbiota members that can access them (5, 6). In addition, dietary components can serve as signaling molecules that regulate the production of specific colonization factors in certain gut commensal species (7). Therefore, the host diet governs the composition of the microbiota by promoting or inhibiting the expansion of specific microbial species in the gut.

Typically associated with healthy individuals, Bacteroides thetaiotaomicron is a gut microbe that devotes ∼18% of its genes to the acquisition and utilization of a wide variety of carbohydrates (8). One of these genes specifies a transcription factor—named BT4338—that is required both for the utilization of multiple polysaccharides and their monosaccharide constituents (9) and for murine gut colonization (10, 11). BT4338 is proposed to bind a DNA sequence present in the putative promoter regions of many genes mediating carbohydrate utilization (9, 12). While these results imply that BT4338 promotes gut colonization by enabling the utilization of specific dietary polysaccharides, a mutant lacking the *BT4338* gene exhibited a similar gut colonization defect regardless of dietary polysaccharides present in the host diet (10). Therefore, BT4338 may control processes necessary for gut colonization but dispensable for carbohydrate utilization.

Protein synthesis requires ribosomes, amino acids, tRNAs, GTP, and several proteins that play distinct roles in the initiation, elongation, and termination of translation as well as the recycling of ribosomes. Elongation factor G (EF-G) is an indispensable GTPase that functions in two different phases of protein synthesis in Escherichia coli and other studied bacteria (13). During translation elongation, EF-G promotes translocation—the movement of the tRNA-peptidyl-tRNA-mRNA complex relative to the ribosome—after a peptide bond has been formed, making the A-site in the ribosome available for decoding the next codon. During ribosome recycling, EF-G works together with the ribosome recycling factor (14, 15) to catalyze the rapid dissociation of the ribosomal post-termination complex into subunits. Some bacterial species, such as E. coli, harbor a single EF-G-specifying gene (designated *fusA*), while others, such as B. thetaiotaomicron, harbor two EF-G-specifying genes (*fusA* and *fusA2*). The deduced amino acid sequences of the B. thetaiotaomicron *fusA* (*BT2729*) and *fusA2* (*BT2167*) genes are 57% and 34% identical, respectively, to the E. coli EF-G protein. Whereas *fusA* is putatively essential, *fusA2* is not (11). However, *fusA2* inactivation compromises gut colonization in multiple *Bacteroides* species (10).

We now report that the master regulator of carbohydrate utilization in *B. thetaiotaomicon*—termed BT4338—controls gut colonization independently of its ability to govern carbohydrate utilization. We identify both the transcriptional profiles of isogenic wild-type and *BT4338*-deficient B. thetaiotaomicron and the sequences bound by the BT4338 protein *in vivo* genome-wide. We determine that BT4338 binds to promoters and regulates the transcription of genes involved in carbohydrate utilization, including one encoding a protein that promotes immunological tolerance in the murine gut. Surprisingly, the mRNA most highly activated by BT4338 corresponds to the *fusA2* gene. We demonstrate that direct transcriptional activation of the *fusA2* gene by BT4338 is essential for gut colonization despite *fusA2* being dispensable for *in vitro* growth on all investigated BT4338-dependent nutrients. In addition, conditions that increase *fusA2* mRNA abundance >200-fold result in a 10-fold decrease in *fusA* mRNA abundance. The transcriptional activation of *fusA2* by BT4338 is conserved in other *Bacteroides* species, suggesting that gut colonization requires specific translation factors.

## RESULTS

### Carbon limitation promotes transcription of the BT4338-activated gene *araM*.

The *BT4338* gene is required for expression of the arabinose utilization gene *araM* (*BT0356*) in B. thetaiotaomicron cultures supplied with arabinose and for growth on arabinose as the sole carbon source (9). Curiously, *araM* is also transcriptionally activated following a shift from growth on glucose to carbon limitation (9) (i.e., in the absence of arabinose), indicating that arabinose is not essential for *araM* transcription.

**FIG 1:**
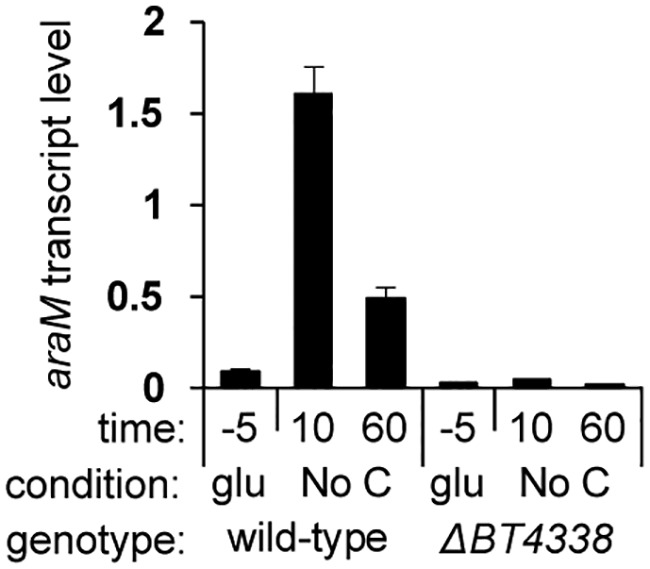
BT4338 transiently activates *araM* transcription during carbon limitation. Shown are *araM* mRNA abundances relative to those of 16S rRNA in wild-type (GT23) and *BT4338* mutant (NS364) B. thetaiotaomicron during mid-log growth in minimal medium containing glucose as the sole carbon source (glu) and 10 and 60 min following a switch to carbon limitation conditions (No C). Measurements were carried out by qPCR and represent averages of 6 independent biological replicates; error bars represent SEM.

We have now determined that the mRNA abundance of the *araM* gene increases 18.1- and 5.6-fold following 10- and 60-min exposures of wild-type B. thetaiotaomicron to carbon limitation, respectively (Fig. 1). By contrast, the *araM* mRNA abundance in the *BT4338* mutant increased <2-fold after 10 min and decreased after 60 min (Fig. 1). These results indicate that *BT4338* is necessary for transient transcriptional activation of the *araM* gene in cells experiencing carbon limitation.

### High-throughput RNA sequencing (RNA-seq) analysis identifies BT4338 as a global regulator.

**FIG 2:**
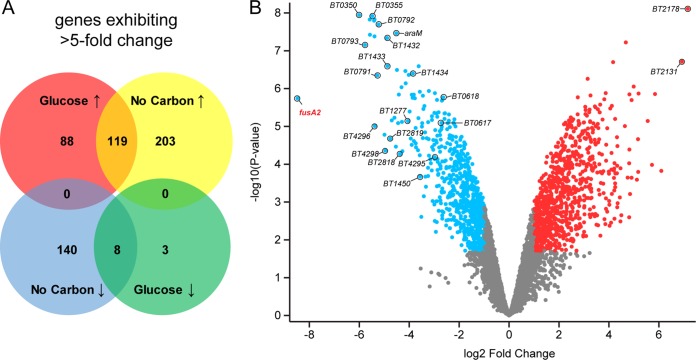
The BT4338 regulon in bacteria experiencing carbon limitation. Shown are the results of RNA-seq analysis of wild-type (GT23) and *BT4338* mutant (NS364) B. thetaiotaomicron during growth in minimal medium containing glucose as the sole carbon source and at 10 min following a switch to carbon limitation conditions. (A) Venn diagrams representing genes whose corresponding mRNAs are >5-fold different between wild-type (GT23) and *BT4338* mutant (NS364) B. thetaiotaomicron. (B) Volcano plot depicting the fold change versus the −log_10_ of the corresponding *P* value for all genes between wild-type (GT23) and *BT4338* mutant (NS364) B. thetaiotaomicron switched from growth in glucose to carbon limitation for 10 min. Blue dots represent genes exhibiting a >2-fold decrease in expression in the *BT4338* mutant compared to that in the wild type. Red dots represent genes exhibiting a >2-fold increase in expression in the *BT4338* mutant compared to that in the wild type. Genes discussed in Results and Discussion are labeled. Data correspond to DESeq2 analysis of RNA-seq analysis of 3 biological replicates examined by featureCounts.

We examined mRNA abundance genome-wide in wild-type and *BT4338* mutant B. thetaiotaomicron at mid-logarithmic phase of growth on glucose (see Fig. S1A and Table S1 in the supplemental material) and after a 10-min exposure to carbon limitation (Fig. 2 and Table S2). During growth on glucose, the mRNA abundance of 11 genes was 5-fold lower, while that of 207 genes was 5-fold higher, in the *BT4338* mutant than in the wild-type strain (Fig. 2A; adjusted *P* values [*P*_adj_] < 0.05; *P* values, determined by analysis of variance [ANOVA], were adjusted using the Benjamini-Hochberg procedure). The numbers of mRNAs whose abundances changed 2-fold are 225 and 832, respectively (Fig. S2B). For example, the mRNA abundance of *BT2131* was 57-fold greater in the BT4338 mutant than in the wild-type strain and represents the greatest increase in transcription resulting from the loss of this transcription factor during growth in glucose (Fig. S1A and Table S1). Conversely, the *BT4543* transcript was 20-fold lower in the *BT4338* mutant than in the wild-type strain under this condition (Fig. S1A and Table S1). These results indicate that BT4338 is necessary for proper transcription of subsets of target genes during mid-exponential-phase growth in glucose, a condition in which the *BT4338* mutant grows similarly to wild-type B. thetaiotaomicron (9).

10.1128/mBio.03221-19.1 FIG S1 The BT4338 regulon during growth in glucose. (A) Volcano plot depicting the fold change versus the −log_10_ of the corresponding *P* value for all genes between wild-type (GT23) and *BT4338*-deficient (NS364) B. thetaiotaomicron strains during growth in glucose. Blue dots represent genes exhibiting a >2-fold decrease in expression in the *BT4338* mutant compared to that in the wild type. Red dots represent genes exhibiting a >2-fold increase in expression in the *BT4338* mutant compared to that in the wild type. Genes discussed in the Results and Discussion sections of the text are labeled. Data represent the averages of 3 biological replicates measured by RNA-seq. (B) Venn diagrams representing genes whose mRNAs are >2-fold different between wild-type (GT23) and *BT4338*-deficient (NS364) strains of B. thetaiotaomicron growing in mid-exponential phase in minimal medium containing glucose as a sole carbon source and at 10 min following a switch to carbon limitation conditions. Data represent the averages of 3 biological replicates measured by RNA-seq, with an adjusted *P* value of <0.05. Download FIG S1, PDF file, 0.2 MB. Copyright © 2020 Townsend et al. 2020 Townsend et al. This content is distributed under the terms of the Creative Commons Attribution 4.0 International license.

10.1128/mBio.03221-19.2 FIG S2 Complementation of a *BT4338*-null mutant with a gene specifying an epitope-tagged BT4338 protein. (A to C) Growth (OD_600_) of wild-type B. thetaiotaomicron harboring an empty vector (GT1009; black lines) or a *BT4338* mutant harboring either the empty vector (NS432; gray lines), a plasmid-encoded wild-type BT4338 (GT1498; blue lines), BT4338 with a C-terminal HA tag (NS433; orange lines), or BT4338 with a C-terminal HA epitope separated by a linker comprised of 4 glycine residues (GT1481; green lines). Bacteria were grown in minimal medium containing either arabinose (A), glucuronate (B), or xylose (C). (D) *fusA2* mRNA abundances in strains GT1009, GT1498, GT1481, and NS432 during growth in glucose (glu, -5) and 10 or 60 min following a switch to carbon limitation conditions (No C). mRNA abundance was measured by qPCR. Data represent the averages of 4 biological replicates, and error bars represent SEM. Download FIG S2, PDF file, 0.3 MB. Copyright © 2020 Townsend et al. 2020 Townsend et al. This content is distributed under the terms of the Creative Commons Attribution 4.0 International license.

10.1128/mBio.03221-19.5 TABLE S1 Genes with significantly altered expression in a BT4338-deficient strain (NS364) relative to that in wild-type B. thetaiotaomicron (GT23) during mid-exponential-phase growth in minimal medium containing glucose. Download Table S1, XLSX file, 0.5 MB. Copyright © 2020 Townsend et al. 2020 Townsend et al. This content is distributed under the terms of the Creative Commons Attribution 4.0 International license.

10.1128/mBio.03221-19.6 TABLE S2 Genes with significantly altered expression in a BT4338-deficient strain (NS364) relative to that in wild-type B. thetaiotaomicron (GT23) following a 10-min exposure to carbon limitation. Download Table S2, XLSX file, 0.5 MB. Copyright © 2020 Townsend et al. 2020 Townsend et al. This content is distributed under the terms of the Creative Commons Attribution 4.0 International license.

After 10 min of carbon limitation, the mRNA abundance of 148 genes was 5-fold lower, while that of 322 genes was 5-fold higher, in the *BT4338* mutant than in the wild-type strain (Fig. 2A; *P*_adj_ values < 0.05; *P* values, determined by ANOVA, were adjusted using the Benjamini-Hochberg procedure). The numbers of mRNAs whose abundances changed 2-fold are 688 and 990, respectively (Fig. S1B). The mRNA abundance of certain genes changed >5-fold both during growth on glucose and upon carbon limitation: 8 genes were lower in the *BT4338* mutant than in the wild-type strain, and 119 were higher in the *BT4338* mutant than in the wild-type strain (Fig. 2A). Below we discuss some of the genes regulated by BT4338 during carbon limitation.

### BT4338-regulated genes participating in carbohydrate utilization and central metabolism.

The *BT4338* gene is required for growth on arabinose, fucose, glucuronate, and xylose (9). Moreover, the BT4338 protein was predicted to bind to the promoters of the genes involved in the utilization of these monosaccharides (9, 12). We have now established that when B. thetaiotaomicron experiences carbon limitation, the mRNA abundance of the genes in the following operons increase in a *BT4338*-dependent manner: the predicted *BT0356-BT0350* operon, which encodes products corresponding to arabinose utilization genes; the *BT1272-BT1277* operon, corresponding to fucose utilization genes; the *BT1434-BT1432* operon, corresponding to glucuronate utilization genes; and the *BT0791-BT0794* operon, corresponding to xylose utilization genes (Fig. 2B and Table S2). These increases took place despite the absence of the specific monosaccharides, the utilization of which requires the activated operons.

Expression of genes required for the utilization of particular polysaccharides is also *BT4338* dependent during carbon limitation (Table S2). These genes include those in the *BT2818-BT2825* locus (*BT2818-BT2822* operon and *BT2823*, *BT2824*, and *BT2825* genes), which is regulated by porcine mucin O-glycans (16); the *BT0453-BT0449* and *BT0439-BT0443* operons, which are induced *in vivo* by unknown substrates (16); and the *BT4299-BT4295* operon, which is induced by *N*-acetyl-d-lactosamine (17). Several proteins are encoded in the *BT4299-BT4295* operon, one of which is implicated in immune cell development (18).

Transcription of genes encoding products predicted to participate in central metabolism and bioenergetics requires BT4338 (Table S2). For example, the *BT1450-BT1448* operon encodes a putative propionyl coenzyme A (propionyl-CoA) carboxylase beta-subunit, biotin carboxylase, and biotin carboxyl carrier protein. These proteins are predicted to form a multi-subunit complex that catalyzes the conversion of propionyl-CoA to d-methylmalonyl-CoA, which is necessary for multiple cellular functions, including acetyl-CoA assimilation in alphaproteobacteria (19), mycolic acid biosynthesis in Mycobacterium tuberculosis (20), and polyketide synthesis in *Streptomyces* species (21).

Additionally, the *BT4338* mutant exhibited a 5-fold decrease in *rnfABCDEG* (*BT0617-BT0622*) mRNA abundance following carbon limitation (Fig. 2B and Table S2). The genes in this operon specify subunits of a protein complex that catalyzes the oxidation of reduced ferredoxin to NAD^+^, which generates an ion gradient across the inner membrane that is harnessed for cellular energy production (22, 23). This finding suggests that BT4338 controls the intracellular pools of NADH/NAD^+^, which are likely altered following a shift from rapid growth in glucose to carbon limitation conditions. In support of this notion, carbon limitation resulted in a 28-fold increase in the *BT1554* mRNA amounts in a BT4338-dependent fashion (Table S2). The *BT1554* gene encodes alanine dehydrogenase, an enzyme that couples the conversion of alanine into pyruvate with the reduction of NAD^+^ into NADH and serves to control the redox state in other bacterial species (24).

Cumulatively, these data indicate that B. thetaiotaomicron responds to carbon limitation by increasing transcription of determinants favoring energy production via the transcriptional regulator BT4338.

### The BT4338 protein binds to the promoter regions of a subset of *BT4338-*activated genes.

**FIG 3:**
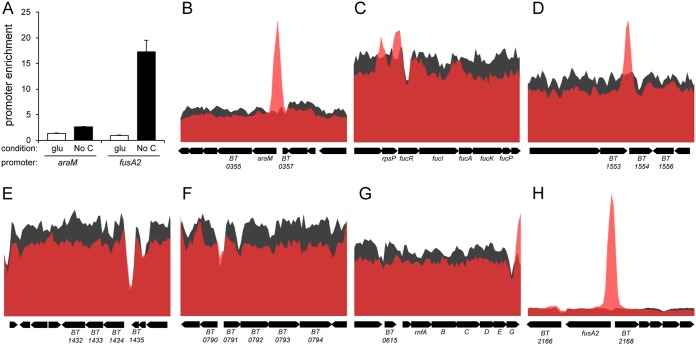
Genome-wide DNA binding by the BT4338 protein. (A) Fold enrichment of the *araM* (*BT0356*) and *fusA2* (*BT2167*) promoter regions during exponential-phase growth in minimal medium containing glucose and following a 10-min exposure to carbon limitation in a strain expressing an epitope-tagged BT4338 protein (GT1481). Measurements were carried out by ChIP-qPCR and represent averages of 4 independent biological replicates; error bars represent SEM. (B to H) BT4338 binding analyzed by high-throughput sequencing (ChIP-seq) upstream of the following genes: the arabinose utilization operon (*BT0356* to *BT0350*) (B), fucose utilization genes (*BT1272* to *BT1277*) (C), glucuronate utilization genes (*BT1434* to *BT1432*) (D), xylose utilization genes (*BT0791* to *BT0794*) (E), the alanine dehydrogenase gene (*BT1554*) (F), *rnfABCDEF* (G), and *fusA2* (*BT2167*) (H). Black represents the input DNA, and red represents the immunoprecipitated DNA. Data represent the normalized abundances of reads mapped across the B. thetaiotaomicron genome and visualized in Artemis.

To determine which of the genes differentially expressed in a *BT4338*-dependent manner following carbon limitation are direct targets of the BT4338 protein, we examined genome-wide occupancy by BT4338 using chromatin immunoprecipitation sequencing (ChIP-seq). Because antibodies directed against the BT4338 protein were not available, we engineered a strain that expressed a C-terminal epitope-tagged BT4338 protein from its normal promoter. This approach was successfully used with other DNA binding proteins (25, 26). The strain expressing the epitope-tagged BT4338 protein supported growth on three different BT4338-dependendent carbohydrates (Fig. S2A to C) and induced *fusA2* expression upon carbon limitation (Fig. S2D). The engineered strain showed ChIP enrichment of the promoter regions of the *araM* and *fusA2* genes during carbon limitation (Fig. 3A), which produced mRNAs in response to carbon limitation in a *BT4338*-dependent manner (Fig. 2B).

BT4338 bound to the promoter regions of the arabinose and fucose utilization genes and the alanine dehydrogenase gene (Fig. 3B to D). By contrast, BT4338 binding was not observed upstream of the glucuronate and xylose utilization genes (Fig. 3E and F), polysaccharide utilization genes, or the *rnfABCDEG* operon (Fig. 3G), even though they are all transcriptionally induced during carbon limitation in a BT4338-dependent fashion (Fig. 2B). These results suggest that BT4338 regulates the latter genes only indirectly, via another regulator. Alternatively, BT4338 binding to the latter genes may require additional signals or be compromised by the epitope at the C terminus of the BT4338 protein used in the ChIP-seq experiments, as this strain does not grow as well as the wild-type parent on xylose and glucuronate (Fig. S2B and C).

Our ChIP-seq analysis revealed that BT4338 binds to 834 locations in the B. thetaiotaomicron genome (Table S3A). These locations correspond to 8 intergenic peaks, 200 intragenic peaks, and 626 peaks that span both intergenic and intragenic regions. BT4338 binding is associated with changes in the mRNA abundances of 175 associated genes, corresponding to 35 BT4338-activated genes and 140 BT4338-repressed genes (Table S3B). Curiously, 184 of 200 intergenic sites, 5 of 8 intragenic sites, and 505 of 626 intra- and intergenic sites bound by BT4338 *in vivo* are not associated with changes in gene expression. (Note that one peak can be associated with multiple genes nearby.) This phenomenon has been reported for other DNA binding regulatory proteins in different bacterial species (27, 28).

10.1128/mBio.03221-19.7 TABLE S3 Regions of the B. thetaiotaomicron genome bound by BT4338 following a 10-min exposure to carbon limitation. (A) All 834 regions bound by BT4338 in response to carbon limitation. (B) BT4338 binding corresponding to BT4338-dependent changes in gene expression during carbon limitation. Download Table S3, XLSX file, 0.4 MB. Copyright © 2020 Townsend et al. 2020 Townsend et al. This content is distributed under the terms of the Creative Commons Attribution 4.0 International license.

### *fusA2* is the BT4338-activated gene most highly induced upon carbon limitation.

**FIG 4:**
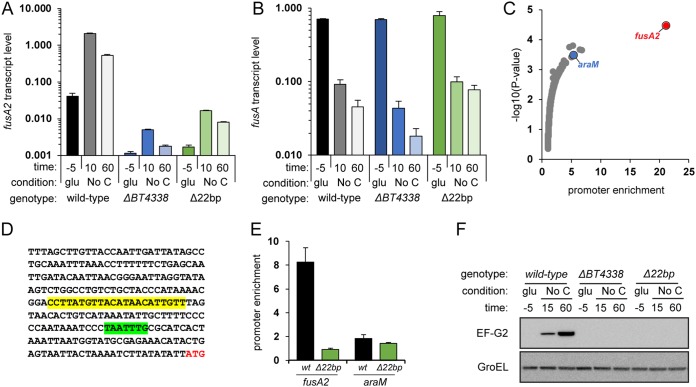
BT4338 directly controls *fusA2* transcription in response to carbon limitation. Shown are *fusA2* (A) and *fusA* (B) mRNAs in wild-type (GT23) and *BT4338* mutant (NS364) B. thetaiotaomicron or a mutant lacking the putative BT4338 binding site in the *fusA2* promoter (WH311) during growth in glucose and 10 min or 60 min following exposure to carbon limitation. mRNA abundance was determined by qPCR. Measurements are averages of 3 independent biological replicates; error bars represent SEM. A log_10_ scale is used for the *y* axis. (C) The fold enrichment versus the −log_10_ of the corresponding *P* value of all regions of the B. thetaiotaomicron chromosome bound by BT4338 following a switch to carbon limitation for 10 min. Enrichment was measured by ChIP-seq. Measurements were made using MACS2 callpeak from 3 independent biological replicates. (D) The DNA sequence upstream of the *fusA2* coding region, with the putative BT4338 binding site highlighted in yellow, the putative −7 consensus sequence highlighted in green, and the start codon lettered in red. (E) Fold enrichment of the *fusA2* and *araM* promoter regions with the BT4338 protein in B. thetaiotaomicron with a wild-type *fusA2* promoter (wt; GT1481) or a strain lacking the 22-bp region (Δ22bp; WH335) highlighted in yellow in panel D. Measurements were carried out by qPCR of ChIP samples and represent averages of 4 independent biological replicates, and error bars represent SEM. (F) Western blot analysis of crude extracts prepared from the wild type (GT1301), a BT4338-deficient mutant (GT1308), and the Δ22bp mutant (WH389) during growth in glucose and 15 or 60 min following exposure to carbon limitation. The top blot was probed for the *fusA2* gene product, EF-G2, and the bottom blot was probed for GroEL.

The largest fold increase in mRNA abundance resulting from a switch from growth in glucose to carbon limitation in wild-type B. thetaiotaomicron (i.e., 238-fold) was in the *fusA2* gene (*BT2167*) (Table S4 and Fig. S3A). *fusA2* mRNA levels increased only 4.7-fold in the *BT4338* mutant (Fig. 4A), while *fusA* mRNA amounts actually decreased 10-fold upon carbon limitation (Table S4 and Fig. S3A), and this decrease was independent of *BT4338* (Fig. 4B). These results indicate that B. thetaiotaomicron coordinates the expression of genes specifying related translation elongation factors in response to carbon limitation.

10.1128/mBio.03221-19.3 FIG S3 B. thetaiotaomicron genes regulated by carbon limitation. (A) Volcano plot depicting the fold change versus the −log_10_ of the corresponding *P* value for all genes in wild-type (GT23) B. thetaiotaomicron in carbon limitation conditions following growth in glucose (Table S4). Blue dots represent genes exhibiting a >2-fold decrease in expression during carbon limitation compared to growth in glucose. Red dots represent genes exhibiting a >2-fold increase in expression during carbon limitation compared to growth in glucose. *fusA*, *fusA2*, and *araM* are labeled. Data represent the averages of 3 biological replicates measured by RNA-seq. (B) *fusA2* mRNA abundances in the wild type (GT23; gray bars) and a strain unable to synthesize (p)ppGpp^0^ (*relA spoT*; GT1181; purple bars) during growth in glucose (-5) and 10 or 60 min following a switch to carbon limitation conditions. mRNA abundance was measured by qPCR. Data represent the averages of 3 biological replicates, and error bars represent SEM. Download FIG S3, PDF file, 0.2 MB. Copyright © 2020 Townsend et al. 2020 Townsend et al. This content is distributed under the terms of the Creative Commons Attribution 4.0 International license.

10.1128/mBio.03221-19.8 TABLE S4 Genes with significantly altered expression in wild-type B. thetaiotaomicron (GT23) following a switch from exponential growth in glucose to carbon limitation conditions for 10 min. Download Table S4, XLSX file, 0.5 MB. Copyright © 2020 Townsend et al. 2020 Townsend et al. This content is distributed under the terms of the Creative Commons Attribution 4.0 International license.

The BT4338 protein governs *fusA2* transcription directly because of the following. First, the *fusA2* promoter region was the most highly enriched DNA region in the ChIP-seq experiment (Table S3A and B, Fig. 3H, and Fig. 4C). Second, a 22-bp region upstream of *fusA2* (CCTTATGTTACATAACATTGTT [Fig. 4D]) resembles the proposed consensus binding sequence for the BT4338 protein (wwwTATGTTnTAnAACATAwww), which is found upstream of many BT4338-activated genes (12). Third, removal of this sequence from the *fusA2* promoter region prevented binding by the BT4338 protein to the *fusA2* promoter, but not the *araM* promoter used as control, under carbon limitation conditions (Fig. 4E). And fourth, the mutant lacking the BT4338 binding site in the *fusA2* promoter exhibited *fusA2* mRNA amounts similar to those produced by the *BT4338*-null mutant under carbon limitation conditions (Fig. 4A). Thus, BT4338 activates *fusA2* transcription by binding to the *fusA2* promoter region.

To determine whether the BT4338 control of *fusA2* mRNA abundance results in changes in EF-G2 protein amounts, we carried out Western blot analysis of crude extracts prepared from isogenic strains expressing an epitope-tagged version of the EF-G2 protein from the normal *fusA2* promoter and chromosomal location. The EF-G2 protein abundance was very low during mid-exponential-phase growth in glucose but rapidly increased following carbon limitation (Fig. 4F). EF-G2 protein amounts did not increase in the *BT4338*-null mutant or in the mutant lacking the predicted BT4338 binding site in the *fusA2* promoter (Fig. 4F).

Cumulatively, the results presented in this section demonstrate that the BT4338 protein directly activates transcription of the *fusA2* gene when bacteria experience carbon limitation.

### (p)ppGpp is dispensable for *fusA2* transcriptional activation during carbon limitation.

We considered the possibility of the alarmone (p)ppGpp controlling *fusA2* transcription because B. thetaiotaomicron produces (p)ppGpp when experiencing carbon limitation (29), and (p)ppGpp represses expression of components of the protein synthesis machinery under this condition (29). However, *fusA2* transcription is (p)ppGpp independent because a mutant unable to make (p)ppGpp due to deletion of the *BT0700* and *BT3998* genes retained wild-type mRNA abundance of the *fusA2* gene (Fig. S3B) (29). These experiments argue that the response to carbon limitation entails (p)ppGpp-dependent and -independent pathways.

### BT4338-dependent transcription of the *fusA2* gene is necessary for gut colonization.

The *BT4338* and *fusA2* genes are both highly conserved across the *Bacteroidetes*, required for gut colonization in three different *Bacteroides* species (10), and transcriptionally upregulated inside the murine gut relative to growth in laboratory media (29). To determine whether BT4338 promotes gut colonization by activating *fusA2* transcription, we examined the abundance of barcoded wild-type B. thetaiotaomicron and mutants lacking either the *BT4338* gene, the *fusA2* gene, or the BT4338 binding site in the *fusA2* promoter in germfree mice inoculated with nearly identical amounts of these strains.

**FIG 5:**
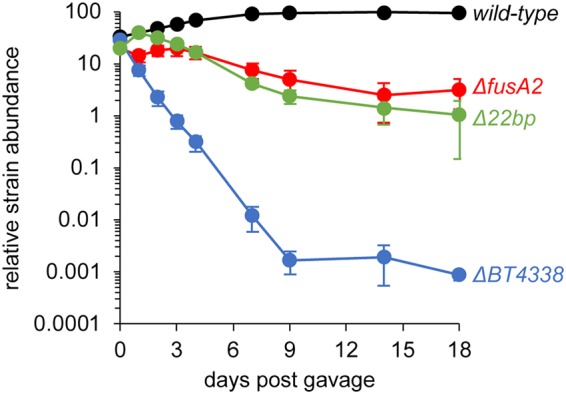
Transcriptional activation of the *fusA2* gene by the BT4338 protein is required for colonization of the murine gut. Shown are the abundances of wild-type B. thetaiotaomicron (GT478), a *BT4338*-deficient strain (WH150), a *fusA2-*deficient strain (WH148), and a strain lacking the 22-bp BT4338 binding site in the *fusA2* promoter (Δ22bp; WH324) at the indicated times after gavage of all four strains into germfree mice. The abundance of each strain was measured by qPCR of unique molecular barcodes. Data represent averages for 5 individual mice, and error bars represent SEM. A log_10_ scale is used for the *y* axis.

Wild-type B. thetaiotaomicron outcompeted all three mutants and became predominant (over 90%) after a 1-week colonization (Fig. 5). These results argue that the colonization defects of the transposon-generated mutants of the *BT4338* gene reported by others (10) are due to loss of function (rather than due to the truncated proteins encoded by the strain with a transposon insertion being toxic to the bacterium during gut colonization). The mutant lacking the BT4338 binding site in the *fusA2* promoter was as defective for gut colonization as that lacking the *fusA2* coding region (Fig. 5), indicating that BT4338 activation of *fusA2* expression is essential for gut colonization. However, the latter two mutants were not as defective as the *BT4338* mutant (Fig. 5), indicating that also BT4338 promotes gut colonization in a *fusA2*-independent manner.

### The *fusA2* gene is dispensable for growth on BT4338-dependent carbon sources.

The B. thetaiotaomicron *BT4338* gene is required for growth on multiple carbohydrates (9), likely due to its critical role in transcriptional activation of the corresponding genes (Tables S1 and S2). By contrast, the *fusA2*-null mutant retained a wild-type ability to grow on carbohydrates that require *BT4338* for growth (Fig. S4). This is surprising because the BT4338 protein exhibited the strongest regulatory effect on the *fusA2* gene (Table S2). These results argue that *fusA2*’s role in gut colonization is unrelated to utilization of the major carbohydrates that require BT4338 for growth *in vitro*.

10.1128/mBio.03221-19.4 FIG S4 *fusA2* is dispensable for growth on BT4338-dependent carbon sources. (A to E) A wild-type B. thetaiotaomicron strain (GT23; black lines) and mutants lacking either *BT4338* (NS364; blue lines) or *fusA2* (GT1309; red lines) were grown in arabinose (A), xylose (B), fucose (C), glucuronate (D), or ribose (E) as a sole carbon source. Data represent the averages of 4 biological replicates and error bars represent SEM. Download FIG S4, PDF file, 0.2 MB. Copyright © 2020 Townsend et al. 2020 Townsend et al. This content is distributed under the terms of the Creative Commons Attribution 4.0 International license.

### *fusA2* regulation by BT4338 sequelogs is conserved among *Bacteroides* species.

The deduced amino acid sequences of the *BT4338* and *fusA2* genes are conserved in many *Bacteroides* species. For example, the BT4338 sequelog in Bacteroides ovatus (encoded by *Bovatus_RS22425*) is 96.1% identical to the *BT4338* gene product and EF-G2 is 97.7% identical between these two species (BT2167 compared to Bovatus_RS10900). Moreover, the *BT4338* and *fusA2* genes are core fitness determinants necessary for gut colonization in three *Bacteroides* species (10). Thus, we wondered whether the transcriptional control of the *fusA2* gene by BT4338 is conserved in other species, given that regulon compositions often differ across related bacterial species (30).

**FIG 6:**
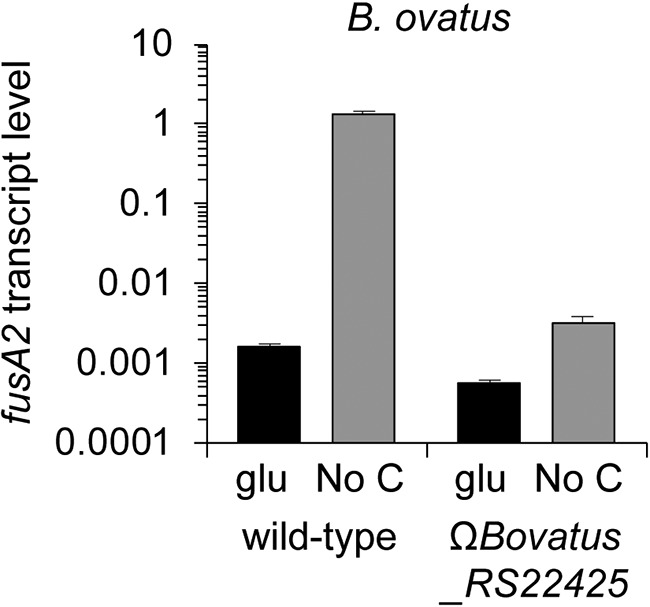
Transcriptional activation of the *fusA2* gene by the BT4338 protein is conserved in Bacteroides ovatus. Shown are *fusA2* (*Bovatus_RS10900)* mRNA abundances in wild-type *B. ovatus* (VR314) and a strain defective for the BT4338 sequelog (Bovatus_*RS22425*; WH275), during mid-log growth in minimal medium containing glucose as the sole carbon source and 10 min following a switch to carbon limitation conditions. mRNA abundance was determined by qPCR. Data represent the averages of 3 independent biological replicates, and error bars represent SEM. A log_10_ scale is used for the *y* axis.

We determined that the abundance of the transcript corresponding to *Bovatus_*RS10900 increased 854-fold following carbon limitation in *B. ovatus* (Fig. 6). This increase is largely dependent on *Bovatus_RS22425* because the *Bovatus_RS10900* transcript increased only 5.2-fold in the *Bovatus_RS22425*-null mutant (Fig. 6). These results indicate that activation of the *fusA2* gene by BT4338 in response to carbon limitation is conserved in *B. ovatus*.

## DISCUSSION

We have now established that the master regulator of carbohydrate utilization in B. thetaiotaomicron controls gut colonization independently of its regulation of carbohydrate utilization genes. We identified the promoter region of the putative translation factor-encoding gene *fusA2* as the DNA most highly bound by BT4338 (Fig. 4C) and the *fusA2* mRNA as the transcript most highly activated by BT4338 (Fig. 2B and Table S2) and the most highly induced upon carbon limitation (Fig. S3A and Table S4). That *fusA* mRNA amounts decrease (Fig. 4B, Fig. S3A, and Table S4) concurrently with an increase in *fusA2* mRNA amounts during carbon limitation (Fig. 4A, Fig. S3A, and Table S4) suggests that B. thetaiotaomicron switches components of its translation machinery during carbon limitation, a condition that bacteria may experience between meals taken by the host. BT4338 control of *fusA2* transcription is conserved in other *Bacteroides* species (Fig. 6). In addition, *fusA2* is dispensable for growth on all nutrients known to require BT4338 (Fig. S4). That transcriptional activation of *fusA2* by BT4338 is critical for B. thetaiotaomicron to colonize the murine gut (Fig. 5) indicates that this master regulator governs gut colonization, in part, by controlling factors putatively responsible for protein synthesis.

### Master regulators of carbohydrate utilization are active during carbon limitation.

Given BT4338’s role as an activator of carbohydrate utilization genes, it is paradoxical that this protein binds to its target DNA sequences during carbon limitation. This binding is physiologically relevant because it results in gene transcription (Fig. 4A), protein production (Fig. 4F), and gut colonization (Fig. 5).

The activity of the master regulator of carbohydrate utilization from E. coli—the cyclic AMP receptor protein (CRP)—is also stimulated by a brief exposure to carbon limitation. In the case of CRP, this is due to the transient synthesis of cyclic AMP (cAMP), the CRP-activating ligand, by adenylate cyclase following a sudden shift from growth in glucose to carbon limitation (31). However, BT4338 does not appear to operate by sensing cAMP produced under carbon limitation conditions because (i) the BT4338 protein exhibits low (i.e., <18%) amino acid identity with CRP, (ii) B. thetaiotaomicron lacks *cya* or *crp* sequelogs, (iii) addition of exogenous cAMP has no consistent effect on gene expression (32), and (iv) the closely related species Bacteroides fragilis encodes a BT4338 sequelog (BF9343_RS04500) but contains no detectable cAMP (33). Thus, BT4338 activates target gene transcription in response to an undefined signal, the availability of which changes in response to carbon limitation and is distinct from cAMP.

### The targets of the master regulator BT4338.

We established that carbon limitation transcriptionally induces both mono- and polysaccharide utilization genes and that this induction is BT4338 dependent. These genes include *BT0356* to *BT0350* (arabinose utilization), *BT1272* to *BT1277* (fucose utilization), *BT1434* to *BT1432* (glucuronate utilization), and *BT0791* to *BT0794* (xylose utilization), as well as *BT2818* to *BT2825*, *BT0453* to *BT0449*, *BT0439* to *BT0443*, and *BT4299* to *BT4295*, which are predicted to mediate polysaccharide utilization (16). These results make sense given that the *BT4338* mutant is defective for growth on various monosaccharides and polysaccharides (9).

Our findings also suggest the intriguing possibility of BT4338 controlling reactions in the tricarboxylic acid (TCA) cycle by regulating acetyl-CoA assimilation in response to various nutrients. This is because the operon encoding the propionyl-CoA carboxylase complex and the gene encoding alanine dehydrogenase are upregulated during carbon limitation in a BT4338-dependent manner.

Our direct experimental approach uncovered hundreds of DNA sequences bound by the BT4338 protein *in vivo*. These sequences are only partly in agreement with those predicted in a bioinformatic study (12) (Table S3A). That is, the bioinformatic study failed to identify many direct targets of BT4338, most conspicuously the *fusA2* promoter, despite being the DNA region most strongly bound by BT4338 *in vivo* (Fig. 3H and Fig. 4C) and harboring sequences that strongly resemble the motif proposed by the bioinformatic analysis (12). The combination of ChIP-seq, RNA-seq, and genetic experiments indicates that the BT4338 protein operates as both a transcriptional activator and repressor.

### Concluding remarks.

The gene most highly regulated by BT4338 during carbon limitation—*fusA2*—specifies a protein that exhibits 31% shared identity (52% similarity) with the deduced amino acid sequence of the B. thetaiotaomicron *fusA* gene, which encodes the canonical elongation factor EF-G. The EF-G and EF-G2 proteins appear to serve different functions in B. thetaiotaomicron because of the following. First, the *fusA* gene is essential under all conditions, whereas *fusA2* is necessary for successful colonization of the murine gut but dispensable otherwise. Second, when *fusA* is highly expressed (i.e., during exponential growth) (Fig. 4B and Table S4), the abundance of *fusA2* mRNA and its protein product, EF-G2, is very low (Fig. 4A and F and Table S4). By contrast, conditions that activate *fusA2* expression, such as carbon limitation (this work) and stationary phase (34), are accompanied by a decrease in *fusA* expression (Fig. 4A and B and Table S4) (35). And third, the increase in *fusA2* mRNA levels taking place during carbon limitation is dependent on BT4338, whereas the concomitant *fusA* mRNA decrease is BT4338 independent.

We suggest that EF-G2 plays two non-mutually exclusive functions in *Bacteroides* species. On the one hand, it provides additional molecules of a translation factor to compensate for the decrease in EF-G abundance. On the other hand, it enables translation of particular mRNAs, perhaps working with ribosomes having a composition distinct from those operating during rapid growth (36). That activation of *fusA2* by BT4338 is required for gut colonization suggests that B. thetaiotaomicron utilizes a distinct protein synthesis machinery when experiencing nutrient fluctuations in its natural habitat.

## MATERIALS AND METHODS

### Bacterial culture conditions.

Bacterial strains and plasmids used in this study are listed in Table S5. E. coli organisms were cultured in Luria-Bertani broth (BD) containing 100 μg/ml of ampicillin (Sigma). B. thetaiotaomicron and *B. ovatus* strains were cultured in brain heart infusion medium containing 5% defibrinated horse blood, tryptone-yeast extract-glucose (TYG) medium, or *Bacteroides* minimal medium [100 mM KH_2_PO_4_ (pH 7.2), 15 mM NaCl, 8.5 mM (NH_4_)_2_SO_4_, 0.5 μg/ml of l-cysteine, 1.9 μM hematin, 200 μM l-histidine, 100 μM MgCl_2_, 1.4 μM FeSO_4_, 50 μM CaCl_2_, 1 μg/ml of vitamin K_3_, and 5 ng/ml of vitamin B_12_, plus individual carbon sources (0.5% wt/vol)] as described previously (9).

10.1128/mBio.03221-19.9 TABLE S5 Strains used in this study. Download Table S5, PDF file, 0.1 MB. Copyright © 2020 Townsend et al. 2020 Townsend et al. This content is distributed under the terms of the Creative Commons Attribution 4.0 International license.

### Construction of strains.

All strains were constructed using oligonucleotides listed in Table S6. In-frame gene deletions and mutations were generated using counterselectable allelic exchange as described (37). Plasmids derived from pNBU2-tetQ were introduced into the B. thetaiotaomicron chromosome (*NBU2 att-1* site) as described previously (37). Molecular barcodes encoded within pNBU2-tetQ vectors were introduced into the B. thetaiotaomicron genome (*NBU2 att-1* site) as described (16). Epitope tagging of *BT2167* (*fusA2*) was performed using the pKNOCK-tetQ vector by cloning the 750-bp sequence at the 3′ end of the *fusA2*, including additional nucleotide sequences at the 3′ end encoding the FLAG tag and a stop codon as described previously (38). Inactivation of the *Bovatus_RS22425* gene was carried out by cloning the sequence from bp 157 to 556 of *Bovatus_RS22425* into the pKNOCK-tetQ vector, followed by its introduction into the *B. ovatus* chromosome as described (38).

10.1128/mBio.03221-19.10 TABLE S6 Oligonucleotides used in this study. Download Table S6, PDF file, 0.1 MB. Copyright © 2020 Townsend et al. 2020 Townsend et al. This content is distributed under the terms of the Creative Commons Attribution 4.0 International license.

### Carbon limitation experiments.

B. thetaiotaomicron and *B. ovatus* strains were grown in TYG medium anaerobically overnight before being subcultured into 2.0 ml of *Bacteroides* minimal medium containing 0.5% glucose as the sole carbon source. After growth to stationary phase, the resulting culture was diluted 1:50 into identical prereduced medium and allowed to grow to mid-exponential phase (optical density [OD] = 0.45 to 0.5), at which time an aliquot was collected by centrifugation for 1 min before decanting and immediate placement of the cell pellet on dry ice until the end of the experiment, which represents measurements designated “-5” in figures for all carbon limitation experiments. Subsequently, the remaining culture was centrifuged at 7,197 × *g* at room temperature for 3 min in sealed tubes and reintroduced into the anaerobic chamber where the tubes were unsealed, and the supernatants were decanted. Cell pellets were resuspended in an equivalent volume of prewarmed, prereduced minimal medium lacking a carbon source and incubated at 37°C anaerobically. At, 10, 15, or 60 min following incubation, aliquots were collected by centrifugation and the supernatant was decanted before the pellet was placed on dry ice prior to storage at –80°C.

### Quantitative PCR (qPCR).

mRNA was prepared from 1.0 ml of B. thetaiotaomicron cell culture treated with RNAprotect (Qiagen) using the RNeasy kit (Qiagen) according to the manufacturer’s instructions. cDNA was subsequently synthesized from 1.0 μg of RNA using Superscript VILO master mix (Thermo Fisher) according to the manufacturer’s directions. The mRNA levels of each gene were measured using a Fast ABI7500 machine (Applied Biosystems) by quantification of cDNA using Fast SYBR green PCR master mix (Applied Biosystems) and primers listed in Table S6. *fusA and fusA2* transcript levels were measured from 10-fold-diluted cDNA using primers W3565 and W3566 or W4344 and W4345, respectively. Data were normalized to 16S rRNA from 1,000-fold-diluted cDNA using primers 10256 and 10257 as described previously (9).

### Western blotting.

Frozen pellets from 12 ml of cells were thawed and resuspended in 400 μl of Tris-buffered saline (TBS; 50 mM Tris, 138 mM NaCl, 2.7 mM KCl [pH 8.0]) containing 1 mM EDTA and 0.5 mg/ml of chicken egg lysozyme (Sigma). Cell suspensions were transferred to a 2.0-ml tube containing lysing matrix B (MP Biomed) and subjected to disruption using a MiniBeadBeater for 5 cycles of 40 s, with 5-min incubations on ice between each cycle. Samples were centrifuged for 2 min at 10,000 × *g* at 4°C to remove cell debris. Protein concentrations were estimated by measuring absorbance at 280 nm using a NanoDrop 8000 (Thermo Fisher). A volume corresponding to 100 μg of protein from each sample was combined with 5 μl of 4× LDS buffer (Thermo Fisher) containing 100 mM dithiothreitol and subjected to heating at 95°C for 5 min. Samples were loaded onto a NuPAGE 4 to 12% bis-Tris protein gel (Thermo Fisher) and fractionated for 60 min at 180 V in 1× morpholinepropanesulfonic acid (MOPS) running buffer (Thermo Fisher). Fractionated proteins were transferred to a nitrocellulose membrane using an iBlot device (Invitrogen), and the resulting membrane was cut below the 65-kDa marker and both portions were blocked for 1 h in TBS containing 3% skim milk. FLAG-tagged EF-G2 were detected on the top portion of the membrane using a 1:5,000 dilution of a mouse anti-FLAG antibody (Sigma) followed by a 1:5,000 dilution of a horseradish peroxidase (HRP)-conjugated anti-mouse antibody (Promega). GroEL was detected on the bottom portion of the membrane using a 1:5,000 dilution of anti-GroEL (Sigma) followed by a 1:5,000 dilution of an HRP-conjugated anti-rabbit antibody (GE). Membranes were washed before and after addition of secondary antibody with TBS containing 0.05% Tween 20 (Sigma) and finally rinsed with TBS prior to detection with Amersham ECL Western blotting detection reagent (GE) using a LAS-4000 (GE).

### RNA-seq.

Ten milliliters of bacterial culture was collected from triplicate cultures growing exponentially in minimal medium containing glucose (OD at 600 nm [OD_600_] = 0.45 to 0.5) or 10 min after centrifugation and resuspension in minimal medium lacking a carbon source. Cell pellets were immediately frozen on dry ice and stored at −80°C. The mRNA was stabilized by treatment with 10 ml of RNAprotect (Qiagen) (diluted 2 parts RNAprotect to 1 part water) prior to extraction using the RNeasy minikit (Qiagen) with on-column DNase I treatment. The eluates were treated with Turbo DNase (Invitrogen) for 30 min before a subsequent round of purification using the RNeasy minikit. RNA-seq was performed at the Yale Center for Genome Analysis, where the RNA quality was examined using a BioAnalyzer (Agilent) before rRNA was depleted using the RiboZero bacterial kit (Illumina). Approximately 50 million 75-bp paired-end reads per sample were collected using a HiSeq 4000 (Illumina).

### ChIP.

A strain expressing a C-terminal hemagglutinin (HA)-tagged BT4338 (GT1481) was grown anaerobically overnight in TYG medium before being subcultured into 2.0 ml of minimal medium containing 0.5% glucose as the sole carbon source. After growth to stationary phase, 2 ml of the resulting culture was inoculated into 100 ml of identical medium and allowed to grow to mid-exponential phase (OD = 0.45 to 0.5). Forty milliliters of cells was combined with 1.08 ml of 37% formaldehyde and gently mixed at room temperature by nutation for 15 min and represented the “-5” time point. Concomitantly, 50 ml of the remaining culture was centrifuged at 7,197 × *g* at room temperature for 3 min. The pelleted cells were resuspended in 50 ml of prewarmed, prereduced minimal medium and incubated at 37°C anaerobically for 10 min before 40 ml of cell culture was combined with 1.08 ml of 37% formaldehyde and gently mixed by nutation for 15 min at room temperature. All cross-linking reactions were halted by the addition of 4 ml of 2.5 M glycine (Sigma), gently mixed by nutation for 5 min at room temperature, and placed on ice until the end of the experiment. Cells were pelleted by centrifugation at 5,000 × *g* for 8 min and washed with 40 ml of ice-cold phosphate-buffered saline (PBS). This wash step was repeated once, and the resulting pellet was resuspended in 1.0 ml of ice-cold PBS, transferred to a 1.5-ml microcentrifuge tube, and pelleted by centrifugation at 5,000 × *g* for 8 min. Cells pellets were immediately frozen on dry ice and stored at −80°C.

The cell pellets were resuspended in 0.5 ml of lysis buffer (10 mM Tris [pH 8.0], 50 mM NaCl, 10 mM EDTA, 20% sucrose) containing 20 mg/ml of lysozyme (Sigma) and protease inhibitor cocktail (Roche). The resuspended cells were combined with 25 μl of RNase A (Qiagen) before incubation at 37°C for 35 min with agitation at 600 rpm in a ThermoMixer (Eppendorf). Following the addition of 80 μl of 10× radioimmunoprecipitation (RIPA) buffer (Millipore) containing protease inhibitor cocktail (Roche), samples were further incubated for 10 min with agitation at 600 rpm in a ThermoMixer (Eppendorf). The samples were cooled on ice prior to DNA fragmentation by sonication at 4°C in a cup horn sonicator (Misonix) with 12- to 15-s pulses at an amplitude of 80, with 30-s rests between each pulse. Debris was pelleted by centrifugation at 18,000 × *g* for 15 min at 4°C, and the supernatant was reserved and stored at −80°C. Upon thawing, 25 μl was reserved as input DNA and the remainder was combined with 10 μl of MagnaChIP protein G beads (Millipore) and incubated at room temperature for 30 min with nutation. The supernatant was recovered and split in half, with one half combined with MagnaChIP protein G beads (Millipore) preincubated with 2 μl of an HA tag-specific antibody (Sigma; IP samples) and the other half combined with MagnaChIP protein G without antibody (control samples). After 2 h of incubation at room temperature with nutation, the beads were recovered using a magnetic stand for 60 s and the bead pellet was washed twice by adding 1 ml of 1× RIPA buffer and gently agitated for 5 min on a nutator. The beads were then washed twice by adding 1 ml of LiCl solution (10 mM Tris [pH 8.0], 250 mM LiCl, 1 mM EDTA, 0.5% Igepal CA-630, 0.5% sodium deoxycholate) and gently agitated for 5 min on a nutator before recovering on a magnetic stand. The beads were then washed twice by adding 1 ml of TE Solution, pH 8.0 (10 mM Tris-HCl, 1 mM EDTA; Millipore) and gently agitated for 5 min on a nutator before recovering on a magnetic stand. Bound DNA was eluted from the beads by the addition of 100 μl of elution buffer (50 mM Tris [pH 8.0], 10 mM EDTA, 1% SDS) and incubation at 65°C for 15 min with mixing at 600 rpm in a ThermoMixer. The beads were collected on a magnetic stand and the supernatant was recovered. The input DNA was brought to 100 μl by addition of 75 μl of elution buffer, and all samples were combined with 100 μl of proteinase K solution and incubated at 37°C for 3 h, followed by 9 h of incubation at 67°C. The DNA was recovered from all input, IP, and control samples using a Qiagen PCR purification kit and eluted in 50 μl of TE buffer. The abundance of the *BT1311* (*rpoD*, for normalization), *araM*, *fusA2*, and *BT3348* promoters were measured in 50-fold-diluted input DNA and 2-fold-diluted IP or control samples using qPCR. The fold enrichment was calculated as described previously (30).

### ChIP-seq.

Triplicate ChIP samples prepared as described above were analyzed by the Yale Center for Genome Analysis, which collected approximately 50 million 75-bp paired-end reads per sampled using a HiSeq 4000 (Illumina).

### High-throughput sequencing data analysis.

The RNA-seq and ChIP-seq alignments and analysis were performed on the Galaxy web server (https://usegalaxy.org). The reads were aligned to the B. thetaiotaomicron genome (strain VPI-5482; GenBank accession number NC_004663) using Bowtie2 (v2.3.4). For RNA-seq samples, the mapped reads were quantified using featureCounts (v1.6.3) and differential gene expression was measured using DEseq2 (v1.18.1). To identify ChIP-seq peaks associated with differential gene expression, the significant ChIP peaks called by MACS2 were assigned as intragenic peaks if they were within annotated genes according to genomic reference NC_004663.1 (GenBank accession number). Conversely, intergenic peaks were defined as those positioned outside annotated coding region.

For analysis of the BT4338 regulon and BT4338-associated regions, genes with a 4-fold (2-log) difference of expression between GT23 and NS364 were selected and assigned with intergenic and/or intragenic peaks that were within a 500-bp distance, if any. For each of the selected genes, the information regarding gene annotation, RNA expression, associated peak location, and associated peak sequences was extracted and reported (Table S3B). The pipeline was written in Python3 with packages pandas, numpy, Biopython, pybedtools, pyBigWig, and pysam. Source code will be made available upon request.

### Examining bacterial abundance in the murine gut.

All experiments using mice were performed using protocols approved by the Yale University Institutional Animal Care and Use Committee. Germfree Swiss Webster mice were maintained in flexible plastic gnotobiotic isolators with a 12-h light/dark cycle and provided a standard, autoclaved mouse chow (5K67 LabDie; Purina) *ad libitum*. Mice were gavaged with around 10^8^ CFU of each strain suspended in 200 μl of phosphate-buffered saline. Input (day 0) abundance of each strain was determined by CFU plating. Fecal pellets were collected at the desired times and genomic DNA was extracted as described previously (16). The abundance of each strain was measured by qPCR, using barcode-specific primers (wild-type strain, primers W1701 and W1713; Δ*BT4338* strain, primers W1704 and W1713; Δ*BT2167* strain, primers W1702 and W1713; and Δ*22* strain, primers W1711 and W1713) as described previously (16).

### Data availability.

The RNAseq data set is available as GEO submission GSE134115. For ChIPseq samples, peaks were called from pooled triplicate input and IP samples using MACS2 (v 2.1.1.20160309), and the corresponding data set is available as GEO submission GSE134116.
