# Erratum for Townsend et al., “A Master Regulator of Bacteroides thetaiotaomicron Gut Colonization Controls Carbohydrate Utilization and an Alternative Protein Synthesis Factor”

**DOI:** 10.1128/mBio.00301-20

**Published:** 2020-03-17

**Authors:** Guy E. Townsend, Weiwei Han, Nathan D. Schwalm, Xinyu Hong, Natasha A. Bencivenga-Barry, Andrew L. Goodman, Eduardo A. Groisman

**Affiliations:** a Department of Microbial Pathogenesis, Yale School of Medicine, New Haven, Connecticut, USA; b Yale Microbial Sciences Institute, West Haven, Connecticut, USA; c Department of Biochemistry and Molecular Biology, Penn State Health Milton S. Hershey Medical Center, Penn State Hershey College of Medicine, Hershey, Pennsylvania, USA

## ERRATUM

Volume 11, no. 1, e03221-19, 2020. https://doi.org/10.1128/mBio.03221-19. On PDF page 3, paragraph 3: “Fig. S2B” should be “Fig. S1B.” On PDF page 6, Fig. 3 legend: “*rnfABCDEF*” should be “*rnfABCDEG.*” On PDF page 8, 9th line from the bottom: “RS10900” should be “*RS10900*”. On PDF page 13: “G.T.” should be “G.E.T.” These changes do not change the conclusions of the paper.

