# Supplementary figures and images for: A Master Regulator of Bacteroides thetaiotaomicron Gut Colonization Controls Carbohydrate Utilization and an Alternative Protein Synthesis Factor
Source: mBio. 2020 Jan 28;11(1):e03221-19. doi: 10.1128/mBio.03221-19 (PMC6989115; doi:10.1128/mBio.03221-19)

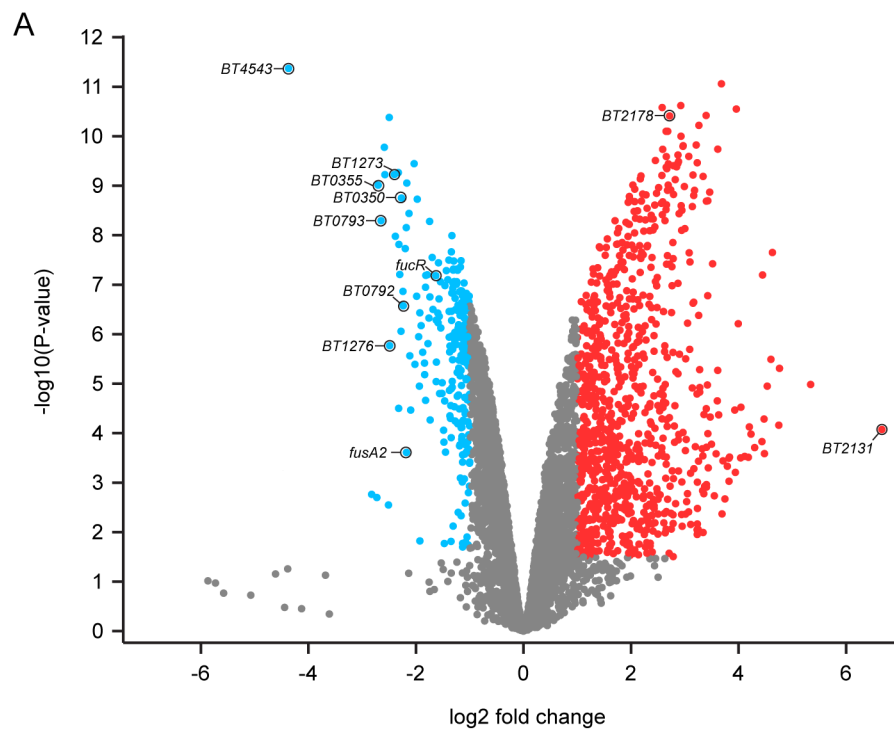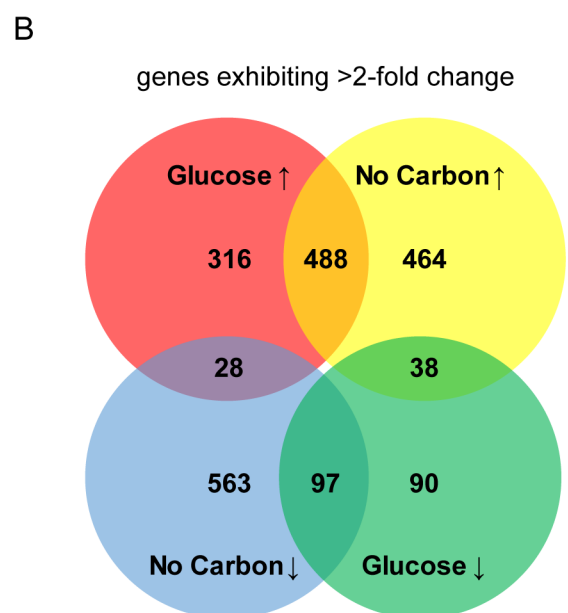

Supplement: FIG S1 [file mBio.03221-19-sf001.pdf]

A

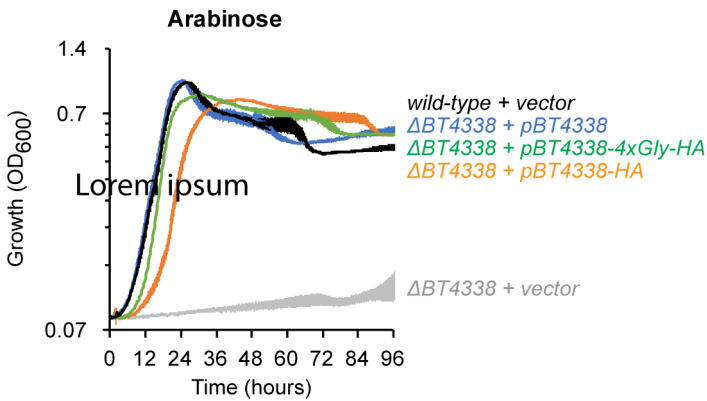

B

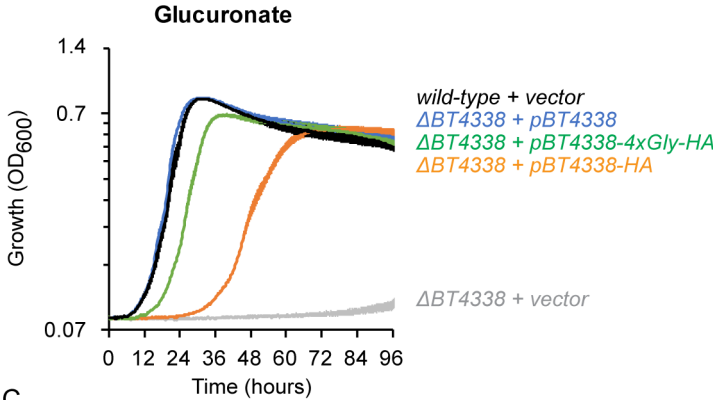

C

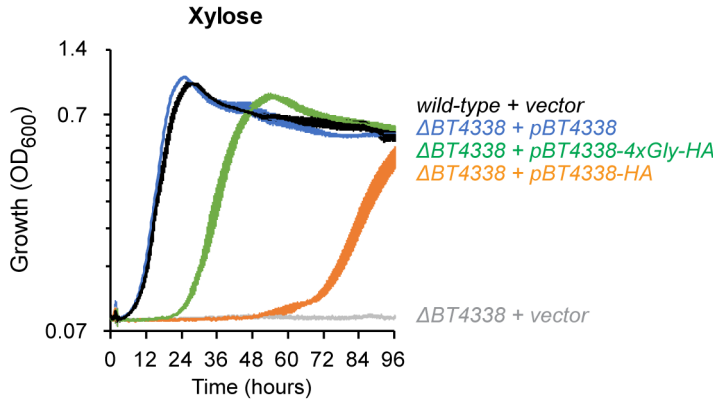

D

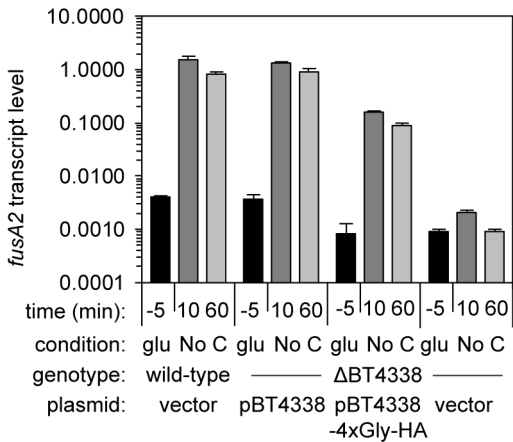

Supplement: FIG S2 [file mBio.03221-19-sf002.pdf]

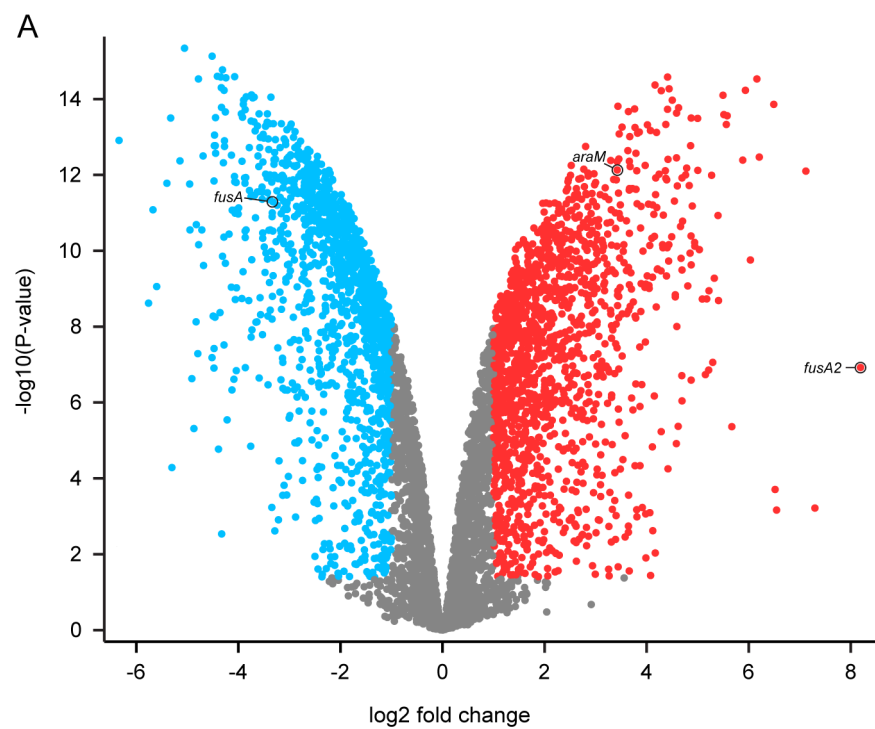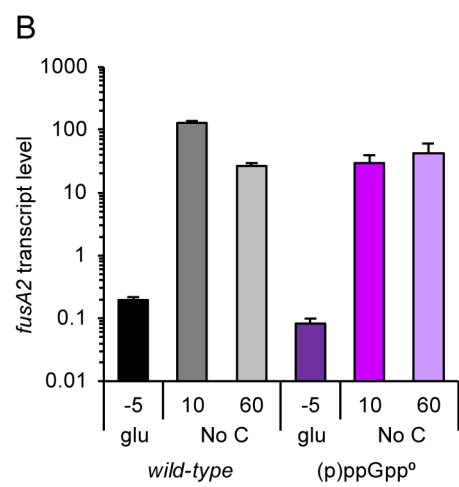

Supplement: FIG S3 [file mBio.03221-19-sf003.pdf]

Lorem ipsum

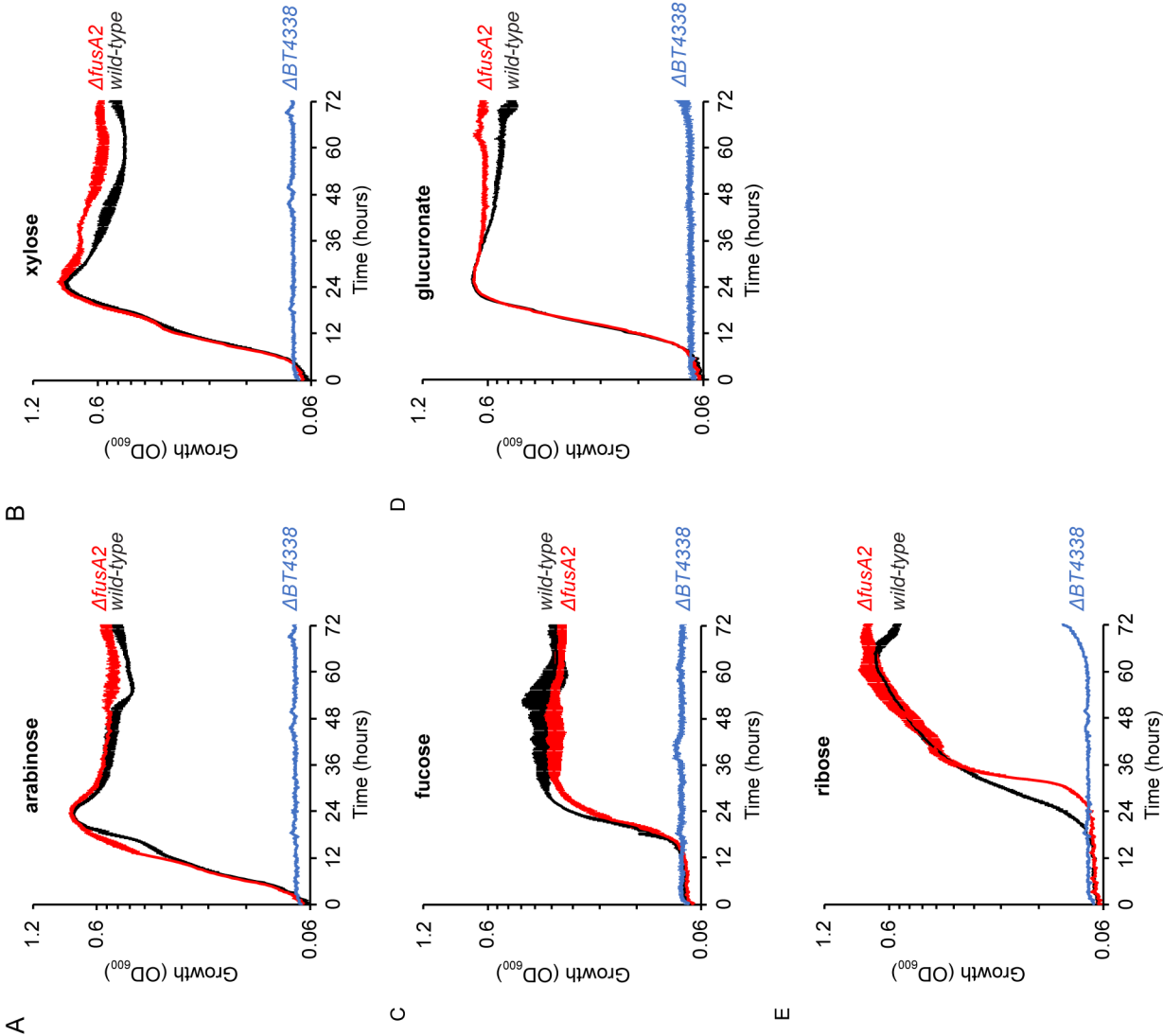

Supplement: FIG S4 [file mBio.03221-19-sf004.pdf]
