# Supplementary material for: A Master Regulator of Bacteroides thetaiotaomicron Gut Colonization Controls Carbohydrate Utilization and an Alternative Protein Synthesis Factor
Source: mBio. 2020 Jan 28;11(1):e03221-19. doi: 10.1128/mBio.03221-19 (PMC6989115; doi:10.1128/mBio.03221-19)
Supplement: TABLE S5 [file mBio.03221-19-st005.pdf]

Table S5. Bacterial strains used in this study.

**Bacterial Strains**	*Source*	*Identifier*
E. coli S17-1 lambda pir	ATCC	ATCC BAA-2428
B. thetaiotaomicron VPI-5482	ATCC	ATCC 29148
B. thetaiotaomicron VPI-5482 *tdk*	PMID: 18611383	GT23
B. thetaiotaomicron VPI-5482 *tdk ΔBT4338*	PMID: 27729509	NS364
B. thetaiotaomicron VPI-5482 *tdk ΔBT2167*	This study	GT1309
B. thetaiotaomicron VPI-5482 *tdk Δ22bp*	This study	WH311
B. thetaiotaomicron VPI-5482 *tdk ΔBT4338 pNBU2-tetQ::P(BT4338)-BT4338-4XGly-HA*	This study	GT1481
B. thetaiotaomicron VPI-5482 *tdk Δ22bp ΔBT4338 pNBU2-tetQ::P(BT4338)-BT4338-4XGly-HA*	This study	WH335
B. thetaiotaomicron VPI-5482 *tdk BT2167-FLAG::pKNOCK-tetQ*	This study	GT1301
B. thetaiotaomicron VPI-5482 *tdk ΔBT4338 BT2167-FLAG::pKNOCK-tetQ*	This study	GT1308

B. thetaiotaomicron VPI-5482 *tdk Δ22bp BT2167-FLAG::pKNOCK-tetQ*	This study	WH389
B. thetaiotaomicron VPI-5482 *tdk pNBU2-tetQ::BC01*	This study	GT478
B. thetaiotaomicron VPI-5482 *tdk ΔBT2167 pNBU2-tetQ::BC03*	This study	WH148
B. thetaiotaomicron VPI-5482 *tdk ΔBT4338 pNBU2-tetQ::BC06*	This study	WH150
B. thetaiotaomicron VPI-5482 *tdk ΔBT22bp pNBU2-tetQ::BC21*	This study	WH324
Bacteroides ovatus NCTC11153	ATCC	ATCC 8483
Bacteroides ovatus NCTC11153 *ΩBovatus_RS22425::pKNOCK-tetQ*	This study	WH275
B. thetaiotaomicron VPI-5482 *tdk pNBU2-tetQ*	This study	GT1009
B. thetaiotaomicron VPI-5482 *tdk ΔBT4338 pNBU2-tetQ*	PMID: 27729509	NS432
B. thetaiotaomicron VPI-5482 *tdk ΔBT4338 pNBU2-tetQ:: P(BT4338)-BT4338-HA*	PMID: 27729509	NS433
B. thetaiotaomicron VPI-5482 *tdk pNBU2-tetQ::P(BT4338)-BT4338*	This study	GT1498
B. thetaiotaomicron VPI-5482 *tdk ΔBT0700 ΔBT3998*	This study	GT1181
