# Supplementary material for: A Master Regulator of Bacteroides thetaiotaomicron Gut Colonization Controls Carbohydrate Utilization and an Alternative Protein Synthesis Factor
Source: mBio. 2020 Jan 28;11(1):e03221-19. doi: 10.1128/mBio.03221-19 (PMC6989115; doi:10.1128/mBio.03221-19)
Supplement: TABLE S6 [file mBio.03221-19-st006.pdf]

Table S6. Oligonucleotides used in this study

**Primers for constructing deletion mutants**		
BT2167_5f – forward primer for the region upstream of *BT2167* (*fusA2*): GCTCTAGAACTAGTGGATCCTTTCGCCGGGCAGTCCGTAA	This study	W3569
BT2167_5r – reverser primer for the region upstream of *BT2167* (*fusA2*): AATATATAAGATTTTAGTAATTACTCAGTATGTTTCTCGC	This study	W3570
BT2167_3f – forward primer for the region downstream of *BT2167* (*fusA2*): TTACTAAAATCTTATATATTACAATAATTCTCTCAAAACTGTTAAAACCG	This study	W3571
BT2167_3r – reverse primer for the region downstream of *BT2167* (*fusA2*): AAGATAACATTCGAGTCGACCGTCTAATCATGTCCAACACTACTTC	This study	W3572
mutP2167uR – reverse primer for the region upstream of the 22bp motif in *BT2167* (*fusA2*) promoter: TATTTATGACAGTGTTACTATCCGTTTTATGGGTAGCAG	This study	W4686
mutP2167dF – forward primer for the region downstream of the 22bp motif in *BT2167* (*fusA2*) promoter: TAGTAACACTGTCATAAATATTGCTTTT	This study	W4687
mutP2167dR – reverse primer for the region downstream of 22bp motif in *BT2167* (*fusA2*) promoter: AAGATAACATTCGAGTCGACACCAATGCTTTATGCATTTC	This study	W4688

**Primers for constructing pNBU2-tetQ plasmids**		
PmalRF – forward primer to generate a construct encoding wild-type BT4338 or BT4338 with a C-terminal epitope tag in pNBU2-tetQ: GCTCTAGAACTAGTGGATCCTCAAAGTACTGGTACGCGAAATGA	PMID: 27729509	W2935
nbu4338-4xG-HAr – reverse primer to generate a construct encoding BT4338 with a C-terminal epitope tag in pNBU2-tetQ: GAAGATAGGCAATTAGTCGACTTAAGCGTAGTCTGGGACGTCGTATGGGTACCCGCCACCTCCTCCTATCTTGCTTATTTCTTGAGTTTTTC	This study	W4101
nbu4338r – reverse primer to generate a construct encoding wild-type BT4338 in pNBU2-tetQ: AAGATAGGCAATTAGTCGACTTATCCTATCTTGCTTATTTCTTGAGTTT	This study	W3185
**Primers for constructing pKNOCK-tetQ plasmids**		
pKOBT2167f – forward primer to generate a construct encoding the C-terminal of BT2167 epitope tag in pKNOCK-tetQ: AGTGGATCCCCGAAGATTCCGTATCGTGA	This study	W3592
nbuBT2167FL – reverse primer to generate a construct encoding the C-terminal of BT2167 epitope tag in pKNOCK-tetQ: AAGATAGGCAATTAGTCGACTTACTTGTCATCGTCATCCTTATAATCTTCCTCTGTTTGTTTAGCTTCAAAATC	This study	W3591
koBo04581f – forward primer to generate a construct encoding the 157-556 bp of BT4338 sequolog in *B. ovatus* (*Bovatus_ RS22425*) in pKNOCK-tetQ	This study	W4645

koBo04581r – reverse primer to generate a construct encoding the 157-556 bp of BT4338 sequolog in *B. ovatus* (*Bovatus_ RS22425*) in pKNOCK-tetQ	This study	W4646
**Primers for measuring gene expression by qPCR**		
qBT16sF – forward primer for measuring the *fusA2* transcript by qPCR:GGTAGTCCACACAGTAAACGATGAA	PMID: 27729509	10256
qBT16sRr – reverse primer for measuring the *fusA2* transcript by qPCR: CCCGTCAAATTCCTTTGAGTTTC	PMID: 27729509	10257
qBT2167f – forward primer for measuring the *fusA2* transcript by qPCR: AAAACGTCGCGGATCTGTTG	This study	W3565
qBT2167r – reverse primer for measuring the *fusA2* transcript by qPCR: TTTCCAGACGCCACTTCAAC	This study	W3566
q2729F890 – forward primer for measuring the *fusA* transcript by qPCR: CGAATACAGGTGCAGAAGAA	This study	W4344
q2729R990 – reverse primer for measuring the *fusA* transcript by qPCR: GTCAAACGACCTACATAAGGG	This study	W4345

qBT0356F – forward primer for measuring the *araM* transcript by qPCR: AACGGCAACGGATATGATCAC	PMID: 27729509	W1784
qBT0356R – reverse primer for measuring the *araM* transcript by qPCR: GCTACCTGCGAGATGTCACCTT	PMID: 27729509	W1785
**ChIP-qPCR primers**		
1311chipF – forward primer for measuring the ChIP-enrichment of *BT1311* promoter: GTCAGTGATCTGGAAGAAGCAATG	PMID: 25041429	13621
1331chipR – reverse primer for measuring the ChIP-enrichment of *BT1311* promoter: GGGAATACACCTGTCAGGAACAA	PMID: 25041429	13622
CHIP2167F1 – forward primer for measuring the ChIP-enrichment of *fusA2* promoter: GCATCACTAAATTAATGGTATGCG	This study	W4852
CHIP2167R1 – reverse primer for measuring the ChIP-enrichment of *fusA2* promoter: CCCAACAAGGCAATGTTCT	This study	W4853

CHIPBT0356F – forward primer for measuring the ChIP-enrichment of *fusA2* promoter: CGGCTTCCGAGCTGACTTTA	This study	W3335
CHIPBT0356R – reverse primer for measuring the ChIP-enrichment of *fusA2* promoter: GGGCATTGTTCACTTCTGTTTG	This study	W3336
3348chipF – forward primer for measuring the ChIP-enrichment of *BT3348* promoter: CTGTGCAAATATGCCCTTCAAG	PMID: 25041429	13313
3348chipR – reverse primer for measuring the ChIP-enrichment of *B3348* promoter: GAATAGACCGTCCCAAATAATCCA	PMID: 25041429	13314
**qPCR primers for bar-coded strains**		
pNBU2_tet_BC01 – forward primer for measuring the bar-code in *pNBU2-tetQ::BC01*: ATGTCGCCAATTGTCACTTTCTCA	PMID: 18996345	W1701
pNBU2_tet_BC03 – forward primer for measuring the bar-code in *pNBU2-tetQ::BC03*: TTATGACCAGCCGCAAATGAAAAG	PMID: 18996345	W1702

pNBU2_tet_BC06 – forward primer for measuring the bar-code in *pNBU2-tetQ::BC06*: GATTACGGCGTGATAGATTGGTGT	PMID: 18996345	W1704
pNBU2_tet_BC21 – forward primer for measuring the bar-code in *pNBU2-tetQ::BC21*: ATGTTCGATCATCAGTTCAGTAGC	PMID: 18996345	W1711
UNIV-R – reverse primer for measuring different bar-code in *pNBU2-tetQ::barcode* plasmids : CACAATATGAGCAACAAGGAATCC	PMID: 18996345	W1713
